# Great Capacity of Physic

**Published:** 1853-09

**Authors:** 


*Great Capacity of Physic.*

THE following has been going the rounds of our exchanges. It is too good to be lost.

"The leading article in a late issue of that brilliant medical journal at 'Keokuk,' is entitled, 'A singular case of continued fever, originating from ulceration of the bowels,' in which the writer introduces his patient in this glorious style: 'His age was six years; temperament of the mixed quality, with redundent shade of the nervo-sanguinous; was inclined to be lean and spare, but possessed a round and muscular fibre.' (?) Two pages of this style intervene, in which we read that the bowels 'evinced normality of function,' and the patient was recommended the 'bain entier' and twenty grains of bi-carbonate of soda, and two grains of sulphate of morphine every three hours!'

Shadrack, Mesheck and Abednego!—but they are a powerful people in Keokuk, both for writin' and physic. If their youngsters stand such treatment, we wonder their daddies ever die. Go away, Fire King!"

